# Identification of SRF-E2F1 fusion transcript in EWSR-negative myoepithelioma of the soft tissue

**DOI:** 10.18632/oncotarget.17958

**Published:** 2017-05-17

**Authors:** Milena Urbini, Annalisa Astolfi, Valentina Indio, Giuseppe Tarantino, Salvatore Serravalle, Maristella Saponara, Margherita Nannini, Alessandro Gronchi, Marco Fiore, Roberta Maestro, Monica Brenca, Angelo Paolo Dei Tos, Gian Paolo Dagrada, Tiziana Negri, Silvana Pilotti, Paolo Giovanni Casali, Guido Biasco, Andrea Pession, Silvia Stacchiotti, Maria Abbondanza Pantaleo

**Affiliations:** ^1^ “Giorgio Prodi” Cancer Research Center, University of Bologna, Bologna, Italy; ^2^ Pediatric Hematology and Oncology Unit, S.Orsola-Malpighi Hospital, University of Bologna, Bologna, Italy; ^3^ Department of Specialized, Experimental and Diagnostic Medicine, Sant'Orsola-Malpighi Hospital, University of Bologna, Bologna, Italy; ^4^ Department of Surgery, Melanoma and Sarcoma Unit, Fondazione IRCCS Istituto Nazionale Tumori, Milan, Italy; ^5^ Unit of Experimental Oncology 1, CRO Aviano National Cancer Institute, Aviano, Italy; ^6^ Department of Anatomic Pathology, General Hospital of Treviso, Treviso, Italy; ^7^ Department of Diagnostic Pathology and Laboratory, Laboratory of Experimental Molecular Pathology, Fondazione IRCCS Istituto Nazionale Tumori, Milan, Italy; ^8^ Cancer Medicine Department, Adult Mesenchymal Tumour and Rare Cancer Medical Oncology Unit, Fondazione IRCCS Istituto Nazionale Tumori, Milan, Italy

**Keywords:** myoepithelial neoplasm, SRF, E2F1, fusion, sarcoma

## Abstract

Myoepithelial neoplasms (MN) are rare and not well-circumstanced entities displaying a heterogeneous spectrum of genetic abnormalities, including EWSR1, FUS and PLAG1 rearrangements. However, in the remaining MN no other fusion gene has been described and knowledge concerning secondary acquired molecular alterations is still poor. Therefore, we screened 5 cases of MN of the soft tissue by RNA sequencing with the aim of identifying novel fusion transcripts.

A novel SRF-E2F1 fusion was detected in two cases: one was negative for other fusions while the other showed also the presence of FUS-KLF17. The fusion was validated through independent techniques and, in both cases, SRF-E2F1 was detected only in a subclone of the tumoral mass. SRF-E2F1 maintained the coding frame, thus leading to the translation of a chimeric protein containing the DNA-binding domain of SRF and the trans-activation domain of E2F1. Moreover, ectopical expression of SRF-E2F1 demonstrated that the chimeric transcript is functionally active and could affect tumor growth.

Occurrence in two cases and biological relevance of the two genes involved suggest that the SRF-E2F1 fusion might become a helpful diagnostic tool. Further biologic studies are needed to better assess its role in MN biology.

## INTRODUCTION

Myoepithelial neoplasms, namely mixed tumor, myoepithelioma (ME) and malignant myoepithelioma/myoepithelial carcinoma (MME), are rare and not well circumstanced entities having similarities to salivary gland counterparts [1] described in soft tissue, skin, bone, breast, kidney [2], thoracic region [3] and vulvar region [4]. The pathologic and molecular characteristic of MN have been better defined only in the last few years.

Given the rarity, the heterogeneous morphology, the poorly informative immunophenotype and the lack of well-defined prognostic criteria, this entity poses diagnostic and clinical challenges. In particular, MN needs to be distinguished from extraskeletal myxoid chondrosarcoma, ossifying fibromyxoid tumors [5], myxoinflammatory fibroblastic sarcoma, chordoma [6], poorly differentiated synovial sarcoma and Ewing sarcoma [7].

Detection of specific fusion transcripts can help to better identify MN and to discriminate them from other entities. According to the literature, nearly half of myoepithelial neoplasm carries *EWSR1* translocations to an ever increasing variety of partners [5, 8]. *EWSR1* is one of the most commonly involved genes in sarcoma translocations. In addition to ME, this gene has been found fused with several distinct partners in a variety of different tumors, including Ewing's sarcoma, extraskeletal myxoid chondrosarcoma, myxoid liposarcoma and others [1, 9]. In addition to *EWSR1*, also *PLAG1* translocations have been described in MN cases with ductal differentiation [10]. Other fusion events were identified in a small subset of myopithelial tumors (6.3%) involving the *FUS* gene [11]. In the remaining fraction of MN, the driver genetic alteration has not been identified yet. Moreover, the role of gene fusions in MN progression remains to be defined.

In 2013, we started an Italian multi-institutional collaboration among centers dedicated to sarcoma with the aim of better defining MN molecular profile. Within this project, still ongoing, we performed whole transcriptome sequencing in five cases, four ME and one MME, to discover novel fusion events.

## RESULTS

### Fusion events prediction

Whole transcriptome sequencing was performed on five tumors: four primary ME and one metastatic MME of the soft tissues (for details see Table 1). Three bioinformatic tools were used for prediction and identification of fusion transcripts. In the two *EWSR1* positive ME tumors it was possible to identify the partners of the fusion: *NFATC2* in one case and *PBX3* in the other. No additional fusion event was detected in these two patients. Instead, the FUS positive case (L108) was predicted to carry a fusion between *SRF* and *E2F1*, besides the primary chimeric transcript involving *FUS* and *KLF17* genes. The same *SRF-E2F1* chimera was also detected in one fusion-negative tumor, L107 (Table 2). All fusion transcripts, either known or novel, were validated by RT-PCR of the breakpoint followed by Sanger sequencing and FISH.

**Table 1:** Clinical characteristic

Pt ID	Gender	Age at disease (years)	Site of primary tumor	Diagnosis	Molecular event	Primary tumor treatment	Local recurrence (Y/N)	Distant recurrence (Y/N)	Disease free survival (months)	Status last follow-up	Overall survival (months)
L107	F	33	iliac region	spindle cell myoepithelioma	Unknown	CSR	N	N	7	NED	7
L108	M	26	right foot	myxed type	FUS translocated	LP + CSR	N	N	60	NED	60
L138	M	43	right arm	malignant myoepithelioma	Unknown	CHT + LP + CSR	Y	Y (lung)	1	DOD	9
L161	M	47	right foot	spindle cell myoepithelioma	EWSR1 translocated	CSR	Y	Y (lymphonodes)	110	NED	324
L162	F	46	Left leg	Myxed type with focal ductal differentiation	EWSR1 translocated	CSR	N	N	54	NED	54

CSR = complete surgical resection, LP= limb perfusion; CHT= Neo-adjuvant CHT

**Table 2:** List of fusion events identified by three bioinformatics predictors: Defuse (Df), ChimeraScan (ChS) and FusionMap (FsM)

Pt ID	5′gene	Breakpoint position 5′gene	3′gene	Breakpoint position 3′gene	Splitted reads	Spanning reads	Frame	Prediction tool
L107	SRF	6:43143805	E2F1	20:32265281	338	632	yes	Df, ChS, FsM
L108	SRF	6:43143805	E2F1	20:32265281	5	25	yes	Df, ChS, FsM
	FUS	16:31198157	KLF17	1:44592015	-	12	yes	ChS
L138	none	-	none	-	-	-	-	-
L161	EWSR1	22:29684775	PBX3	9:128697751	6	43	yes	Df, ChS, FsM
L162	EWSR1	22:29678546	NFATC2	20:50133494			yes	ChS, FsM

### Characterization of the novel SRF-E2F1 fusion

The *SRF*-*E2F1* chimeric transcript was shown to be highly expressed in sample L107, as suggested by the amount of supporting reads (respectively 338 splitted and 632 spanning reads across the junction), while it was less represented in the *FUS*-*KLF17* positive sample (5 split and 25 spanning reads) (Table 2).

**Figure 1:**
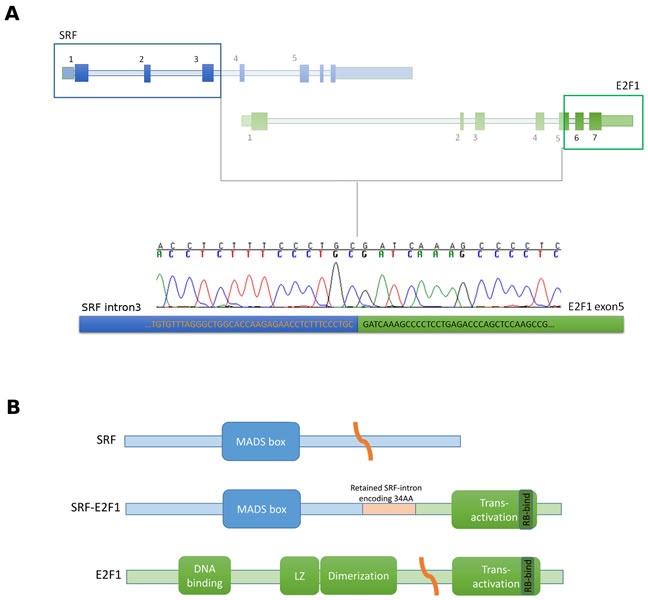
SRF-E2F1 fusion transcript identified by whole-transcriptome sequencing **A.** Schematic representation of *SRF-E2F1* chimeric transcript showing the exact breakpoint sequence identified and validated by Sanger sequencing. **B.** Schematic representation of the protein domains involved in the putative chimeric protein.

*SRF*-*E2F1* was predicted to retain the first 100 bases of the third intron of *SRF* joined with the second half of exon 5 of *E2F1*, exactly 66 bases after the original splice acceptor site (Figure 1A). Despite the complex breakpoint, with the joining of *SRF* intron with *E2F1* exon 5, the fusion transcript maintained the coding frame, thus leading to the translation of a putative chimeric protein. The predicted SRF-E2F1 fusion protein retains the MAD box domain of SRF and the trans-activation domain of E2F1, suggesting that the chimeric protein retains the DNA binding specificity of SRF while adding the trans-activation activity of E2F1 (Figure 1B).

**Figure 2:**
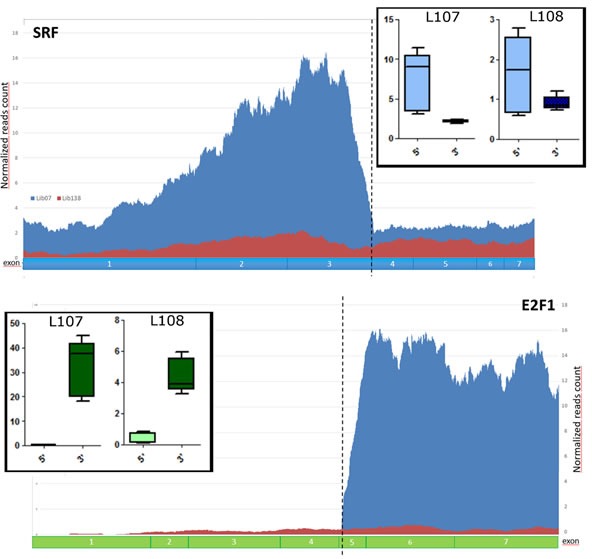
Validation of SRF-E2F1 fusion in L107 and L108 ME tumors Normalized reads count showing higher expression of *SRF* and *E2F1* exons involved in the fusion. In the two larger panels are represented the normalized reads count, respectively in blue for L107 and in red for L138, a sample used as control without *SRF-E2F1* fusion. In the two box-plot are represented the average expressions of *SRF* and *E2F1* exons before (5′) and after (3′) the breakpoint in both L107 and L108.

In both L107 and L108, *SRF* and *E2F1* exons involved in the fusion gene were highly expressed when compared to the ones not participating in the chimera (Figure 2). These data suggest that most of *SRF* and *E2F1* expression in these samples are derived from the fusion transcript rather than from the uninvolved alleles.

In order to identify the genomic breakpoint, we performed PCR amplification also on genomic DNA extracted from the two tumor tissues. PCR amplification of the breakpoint region yielded a PCR product of different size in the two samples, about 300bp for L107 and about 200bp for L108. Sanger sequencing of these amplicons revealed that, in both samples, the genomic breakpoint matched exactly with the *SRF*-*E2F1* junction detected at transcriptional level. In L108 a genomic deletion occurred downstream the breakpoint, involving entirely intron 5 (95bp) and the first 9 bases of exon 6 of *E2F1*, thus leading to the shorter amplicon while keeping the coding frame of *E2F1* (see supplementary File 2).

**Figure 3:**
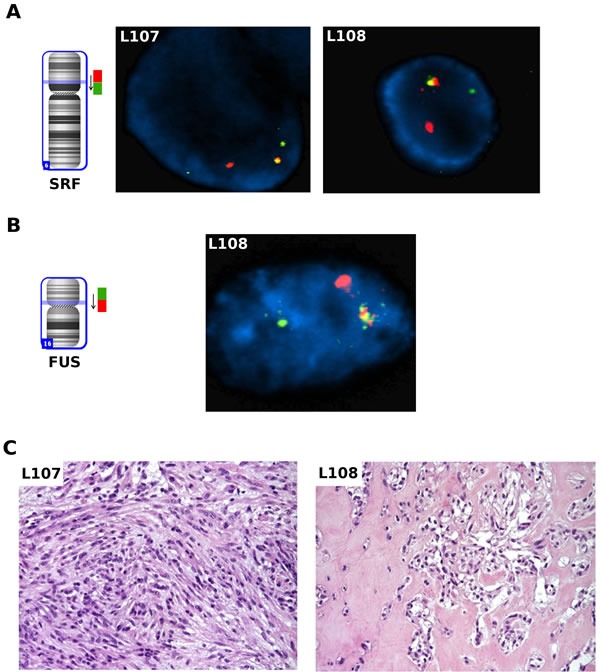
FISH analysis on thawed frozen tumor specimens **A.** FISH analysis for the SRF gene on thawed frozen tumor specimens of L107 and L108: fusion positive cells showed one orange/green fusion, one orange, and one green signal pattern indicative of a rearrangement of one copy of the SRF gene. **B.** FISH analysis for the *FUS* gene on thawed frozen tumor specimen of L108: fusion positive cells showed a signal pattern consisting of one orange/green fusion signal, one orange signal, and a separate green signal indicates one normal 16p11.2 locus and one 16p11.2 locus affected by a *FUS* translocation. **C.** Pathologic findings on the two *SRF-E2F1* positive cases: L107 showed a myoepithelioma comprised of predominantly myoepithelial spindle cells; L108 showed an area of epithelioid cells arranged in nested pattern and embedded in myxoid-hylinized stroma in an otherwise mixed myoepithelioma (not shown).

Then, we performed a FISH break-apart assay on the *SRF* and *FUS* loci to support the presence of the rearrangements through an independent technique and to analyze the clonality of the fusion events (Figure 3A, 3B). In both L107 and L108, corresponding to one spindle cell and one mixed type myoepithelioma respectively (Figure 3C), the rearrangement of *SRF* gene was observed in about 20% of tumor cells. Interestingly, in L108 it was also possible to see the subclonality of this event: while the *SRF* rearrangement was observed only in a subpopulation of the cells, *FUS* rearrangement was observed in almost all tumor cells (92%). Moreover, concomitant presence of both *SRF* and *FUS* rearrangements were observed in the same cell, as shown by re-hybridizing *SRF* FISH slide with the *FUS* break apart probe. Interestingly, the two SRF-E2F1 positive patients were younger than the other three cases with a mean age at diagnosis of 29.5 vs 45.3 years (P < 0.05), even if the cohort analyzed was too small to draw any definitive conclusion.

We then searched, through whole exome sequencing, the presence of other genomic aberrations in the two SRF-E2F1 positive cases. No relevant copy number alteration was identified. A mean of 11 somatic mutations were detected, no recurrently altered gene was present and all mutations were classified as passenger (Supplementary File 3).

**Figure 4:**
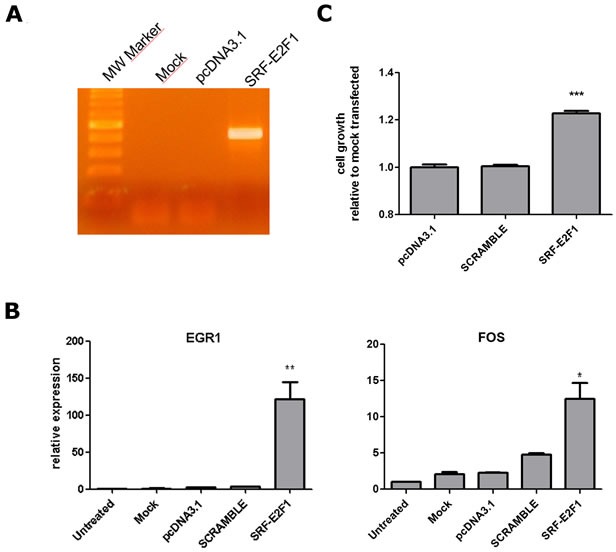
SRF-E2F1 expression in HEK293 cell line **A.** RT-PCR showing *SRF-E2F1* mRNA expression in HEK293 cells, 72h after transfection with *SRF-E2F1* plasmid. Mock (lipofectamine only) and pcDNA3.1 (empty vector) transfected samples are shown as negative controls. **B.** mRNA relative expression of *EGR1* and *FOS*, two genes target of *SRF*, 72h after transfection. Fold changes were evaluated in comparison to untreated sample. *GAPDH* and *GUSB* were used as housekeeping genes. *P* value was estimated against pcDNA3.1 and Scramble using t-test (*p < 0.05; **p < 0.01). **C.** HEK293 relative cell growth evaluated using WST1 assay, 72h after transfection. SRF-E2F1 transfetcted cells showed a significant (***p < 0.001) increase of cell growth in comparison with both scramble and pcDNA3.1 treated cells. P-value was estimated with t-test.

Finally, to assess if *SRF*-*E2F1* could be functionally active, the fusion gene was cloned into a plasmid vector and expressed in HEK293 cell lines (Figure 4A). Expression of the fusion protein led to a marked up-regulation of *EGR1* and *FOS* (Figure 4B), two genes target of *SRF* transcriptional regulation [12], and to a significative increase of cell growth rate with respect to negative controls (22%) (Figure 4C), including a scramble *SRF*-*E2F1* chimera carrying a premature stop codon.

## DISCUSSION

In this work, we reported the identification of a fusion event involving *SRF* and *E2F1* genes in two MN of the soft tissues. This event was detected in one *FUS*-*KLF17*-translocated ME tumor and in one ME negative for fusion transcripts involving either *EWSR1* or *FUS*, both lacking pathological evidence of malignancy according to the criteria outlined in Jo et al [1]. In both tumors, the translocation appeared to be subclonal thus suggesting that it could be acquired over time.

Myoepithelial neoplasms of the soft tissues are very rare tumors characterized in the late '90 that have been recently classified by C. Fletcher into 3 different groups called mixed tumor, myoepithelioma and malignant myoepithelioma/myoepithelial carcinoma [1]. This family of neoplasms described at different anatomic sites [2–4] are marked by a high degree of heterogeneity with respect to both the morphologic and the molecular clues. Furthermore, it is still not completely clear to which extent MN arising from different locations do represent the same entity and only little data are currently available in both MN natural history and sensitivity to medical agents.

It is well known that gene fusions are a relevant class of “driver mutations” in cancer, particularly in hematologic malignancies and in a subset of sarcomas. Several fusion transcripts had been detected in MN, involving *EWSR1*, *PLAG1* and *FUS* [5, 10, 11, 13], however for a significant portion of this tumors no fusion event have been identified and, to date, no reports of secondary acquired fusion genes in this disease are available. The involvement of fusion genes in MN progression is poorly defined.

Here, we report a novel fusion event, consisting in a *SRF*-*E2F1* fusion in two cases of soft tissue ME, one mixed type tumor and one spindle cell myoepithelioma, respectively. Interestingly, the two *SRF*-*E2F1* positive patients were younger respects to the other three cases, however the cohort analyzed was too small to draw a definite conclusion. Moreover, excluding the fusion genes identified, no other relevant alterations were detected through genomic analysis, revealing a generally normal karyotype with few passenger mutations. *SRF*-*E2F1* is a complex fusion that originates from a balanced translocation between chr6 and chr20. Even if the breakpoint was located between the middle of intron 3 of *SRF* and the middle of exon5 of *E2F1*, the predicted chimeric protein retained the coding frame and the critical domains of both proteins. It is not infrequent to detect fusion genes with complex breakpoints similarly to the cases described here, for example some forms of BCR-ABL could have the fusion junction located within coding-exons or could contain intronic regions while maintaining the reading frame [14].

*SRF* encodes a MADS-box transcription factor that, through the binding to CArG box motifs, controls the expression of a wide set of genes including immediate early genes, like *c-Fos*, *Jun* and *Egr*, as well as tissue-specific genes involved in cell proliferation, migration, angiogenesis cytoskeletal organization, energy metabolism and myogenesis [12, 15, 16]. Accumulating evidence suggested that *SRF* may play multiple roles in carcinogenesis and tumor progression in various cancers, specifically in the mesenchymal transition of epithelial tumor cells [17–19]. Recently, fusion genes involving *SRF* were detected in some types of mesenchymal tumors, including *SRF*-*NCOA2* in one case of infantile spindle cell rhabdomyosarcoma [20] and *SRF*-*RELA* in 7 cases of myofribroma and myopericytoma [21]. On the other side, *E2F1* belongs to the E2F family of transcription factors that is involved in cell cycle progression and apoptosis induction in response to DNA damage [22]. *E2F1* plays a critical role in the malignant phenotypes of some cancers. Yet, deregulation of *E2F1* expression can either promote or inhibit tumorigenesis depending on the nature of the cellular context [22, 23].

To our knowledge, fusion events involving *E2F1* has not been previously reported in tumors, however several chimeric proteins has been designed and tested *in vitro* for functional studies. In particular, it was shown that the acidic activation domain of E2F1, located near the C-terminus of the protein, is able to function even when attached to the DNA-binding domain of a heterologous protein [24, 25]. Herein, we demonstrated that the expression of *SRF*-*E2F1* fusion gene produced a chimeric protein functionally active. The role of this novel fusion protein is left to be clarified. However, even if preliminary, these results indicated that the chimeric protein could retain DNA binding specificity of SRF and through the trans activation domain of E2F1 is able to activate SRF target genes, probably supporting tumor growth.

Although *SRF*-*E2F1* was confirmed both at transcriptional and at genomic level, only a subclone of the tumors carried the genomic translocation as shown by FISH. Moreover, the concomitant presence of two fusion events in one tumor sample (*SRF*-*E2F1* and *FUS*-*KLF17* in sample L108), suggests that ME could have heterogeneous composition, with expansion of several clones and accumulation of secondary alterations. Together, these data suggest that *SRF*-*E2F1* fusion could be a secondary event acquired during tumor clonal genetic evolution. Whether the *SRF*-*E2F1* clone has the potentiality of expansion to become the prevalent component of the tumor, thus affecting patient outcome, remains to be elucidated.

This cytogenetic abnormality might become a helpful diagnostic tool and further biologic studies are needed to elucidate its role in MN. The biological relevance of the two genes involved and the fact that this event was found to occur in two different patients, suggest that *SRF*-*E2F1* fusion could be relevant in the tumor natural history, being a late event in the tumor biology time course. It certainly warrants further investigations across larger series.

In conclusion, MN are characterized by an extreme heterogeneity in morphological immunohistochemical and genetic features. In particular, an ever increasing number of different gene fusions is reported in this entity. Here we describe an intra-tumoral heterogeneity which adds a further layer of complexity and suggests that ME could bear multiple driving events, among which different gene fusions.

## MATERIALS AND METHODS

### Case selection

Cases were selected among those operated from 2012 at Fondazione IRCCS Istituto Nazionale Tumori, Milan, Italy, for primary tumor, with diagnosis of MN, arising from the soft tissue, whose fresh/frozen tumor tissue adequate for the analysis was available. Diagnosis was confirmed by sarcoma expert pathologists (SP; APDT). Whole transcriptome sequencing of tumor samples was performed at “Giorgio Prodi” Interdepartmental Center of Cancer Research, University of Bologna, Italy.

This study was approved by the local Ethical Committee of all the involved Institutions.

### Patients and tumor samples

Fresh tissue specimens of five cases of soft tissue MN (2 *EWSR* positive; 1 *FUS* positive; 2 negative for *EWS*, *FUS*, *NR4A3* and *PLAG1*) were collected snap-frozen in liquid nitrogen and stored at −80°C until analysis. Patient characteristics are listed in Table 1.

### Whole-transcriptome sequencing

Total RNA was isolated from fresh frozen tumor tissues using the RNeasy spin-column method (Qiagen, Milan, Italy). Whole-transcriptome RNA libraries were prepared in accordance with Illumina's TruSeq RNA Sample Prep v2 protocol (Illumina, San Diego, California). Briefly, poly(A)-RNA molecules from 500 ng of total RNA were purified using oligo-dT magnetic beads. Following purification, the mRNA was fragmented and randomly primed for reverse transcription followed by second-strand synthesis to create double-stranded cDNA fragments. These cDNA fragments went through a terminal-end repair process and ligation using paired-end sequencing adapters. The products were then amplified to enrich for fragments carrying adapters ligated on both ends and to add additional sequences complementary to the oligonucleotides on the flow cell, thus creating the final cDNA library. 12pM paired-end libraries were amplified and ligated to the flowcell by bridge PCR, and sequenced at 2×75bp read length, using Illumina Sequencing by synthesis technology. An average of 77 million reads were obtained for whole transcriptome analysis. Reads were mapped on the human reference genome by TopHat/BowTie pipeline. For gene fusions discovery, three bioinformatics tools were used: DeFuse, ChimeraScan, and FusionMap. For gene expression evaluation, normalized read counts were determined for each coding position of *SRF* and *E2F1* mRNA, then the ratio between average expressions of exons before and after breakpoint was calculated for each genes.

### Fusion validation

500ng of total RNA was retrotranscribed to cDNA using First Strand cDNA Sythesis kit (Roche) and oligo-dT primers, then the fusion transcript was amplified using primers pair specific for the breakpoint region. Additionally, amplification of the breakpoint was performed also on tumor DNA. After quality check on agarose gel, amplicons were purified and sequenced using the Sanger method. The primers pairs used to amplify and sequence the fusion transcripts of interest were: *SRF*_exon3_Fw 5′-TCACCAACTACCTGGCACCA-3′ and *E2F1*_exon6_Rev 5′-ACATCGATCGGGCCTTGTTT-3′; *FUS*_ex6_Fw 5′- GCTATGGACAGCAGGACCGT - 3′ and *KLF17*_ex2_Rev 5′- GCTGTGAGGAAAGTGCTGAATG -3′

### FISH

FISH analysis was performed on frozen tumor tissue imprints to confirm rearrangements of the *SRF* and *FUS* genes, using two *SRF* specific BlueFISH probes (BlueGnome Ltd., Cambridge) and the ZytoLight SPEC *FUS* Dual Color Break Apart Probe (ZytoVision GmbH, Germany). In detail, for the SRF gene, two BAC clones partially overlapping on the *SRF* gene: RP11-387M24 (extending towards 3′ end) and RP11-480N24 (extending towards 5′end), labelled in red and green respectively, were used.

### Whole exome sequencing

Genomic DNA was extracted from fresh-frozen tumor specimens and from matched PB with QiAmp DNA mini kit (Qiagen). Libraries were synthesized with Nextera Rapid Capture Exome Kit (Illumina) following manufacturer's recommendations. Reads were mapped with BWA and GATK and Mutect were used to call the Ins/del variants and Single nucleotide variants. Somatic mutations were identified by verifying the presence of alternate allele in the normal counterpart. All variants were filtered in order to select novel or rare events (databases: dbSNP, 1000Genomes, ExAC and EVS) and their effect on protein structure and function was predicted with SNPeff. Moreover, copy number analysis (detection of large amplifications or deletions) was performed making a consensus between two softwares (Control FREEC and ADTEX) with paired tumour/matched normal samples. Variations was filtered also on the polymorphic copy number variants from the Database of human Genomic Variants.

### Cloning and transfection of SRF-E2F1 in HEK293 cell line

The entire coding sequence of SRF-E2F1 fusion gene was amplified from cDNA of L107 using the following primers: SRF_Fw 5′-CGCCATGTTACCGACCCAA-3′ and E2F1_Rev 5′- AGAGACAAGGTGAGCATCTCTGG -3′. The amplicon was then cloned into a plasmid vector using pcDNA3.1 TOPO TA Expression kit (Life Technologies). Plasmid DNA of several clones were checked for insert orientation and for mutations through Sanger sequencing. Two clones, one containing an intact *SRF*-*E2F1* and a second one with a premature stop codon (scramble *SRF*-*E2F1*), were selected for subsequent transfection experiments (see supplementary File 1 for scramble sequence). HEK293 cell line were grown on DMEM medium with 10% of foetal bovine serum, 1% L-glu and 1% pen-strep. Cells were transfected with *SRF*-*E2F1*, scramble or empty pcDNA3.1 vector using Lipofectamine 2000 (Life Technologies). Cells untreated and mock (treated with only Lipofectamine) were used as controls. Total RNA was extracted 72h after transfection, and fusion gene expression was evaluated with RT-PCR. mRNA expression level of *EGR1* and *FOS* were evaluated in with realtime PCR (LightCycler480, Roche). To determine the proliferation activity, transfected cells were seeded in triplicate in a 96well plate (10.000 cell/well) and WST1 assay (Roche) was performed 72h after transfection. These experiments were repeated in triplicate. Relative cell growth rate was calculated in comparison with scramble and pcDNA3.1 treated cells. GraphPad PRISM Software was used for graph design and statistical analysis.

## SUPPLEMENTARY FILES



## Abbreviations

MN: Myoepithelial neoplasms
ME: myoepithelioma
MME: malignant myoepithelioma/myoepithelial carcinoma
