# Supplementary material for: Identification of SRF-E2F1 fusion transcript in EWSR-negative myoepithelioma of the soft tissue
Source: Oncotarget. 2017 May 17;8(36):60036–45. doi: 10.18632/oncotarget.17958 (PMC5601120; doi:10.18632/oncotarget.17958)
Supplement: Supplementary file 1 [file oncotarget-08-60036-s001.pdf]

Identification of SRF-E2F1 fusion transcript in EWSR-negative myoepithelioma of the soft tissue

Supplementary Material

Supplementary File 1: Sequence of scramble SRF-E2F1 cloned into pcDNA3.1 plasmid.
Bases corresponding to SRF and E2F1 coding sequences are respectively reported in black and in green. In red is shown the insertion of an "A" that caused a premature stop codon of the fusion protein in the scramble plasmid.

ATGTTACCGACCCAAGCTGGGGCCGCGGCGGCTCTGGGCCGGGGCTCG-
GCCCTGGGGGGCAGCCTGAACCGGACCCCGACGGGGCGGCCGGGCGGCGGC-
GGGACACGCGGGGCTAACGGGGGCCGGGTCCCCGGGAATGGCGCGGGGCTCGGGCCC-
GGCCGCCTGGAGCGGGAGGCTGCGGCAGCGGCGGCAACCACCCCGGCGCCCACC-
GCGGGGGCCCCCTACAGCGGCAGCGAGGGCGACTCGGAGTCGGGCGAGGAGGAG-
GAGCTGGGCGCCGAGCGGCGCGGCCTGAAGCGGAGCCTGAGCGAGATGGAGATCG-
GTATGGTGGTCGGTGGGCCCGAGGCGTCGGCAGCGGCCACCGGGGGCTACGGGCCG-
GTGAGCGGCGCGGTGAGCGGGGCCAAGCCGGGTAAGAAGACCCGGGGCCGCGTGAA-
GATCAAGATGGAGTTCATCGACAACAAGCTGCGGCGCTACACGACCTTCAGCAAGAG-
GAGGACGGGCATCATGAAGAAGGCCTATGAGCTGTCCACGCTGACAGGGA**A**CACAGGT-
GCTGTTGCTGGTGGCCAGTGAGACAGGCCATGTGTATACCTTTGCCACCCGAAAACTG-
CAGCCCATGATCACCAGTGAGACCGGCAAGGCACTGATTCAGACCTGCCTCAACTCGC-
CAGACTCTCCACCCCGTTCAGACCCCACAACAGACCAGAGAATGAGTGCCACTGGCTTT-
GAAGAGACAGATCTCACCTACCAGGTGTCGGAGTCTGACAGCAGTGGGGAGACCAAGGA-
CACACTGAAGCCGGCGTTCACAGTCACCAACCTGCCGGGTACAACCTCCACCATC-
CAAACAGCACCTAGCACCTCTACCACCATGCAAGTCAGCAGCGGCCCCTCCTTTCCCAT-
CACCAACTACCTGGCACCAGTGTCTGCTAGTGTCAGCCCCAGTGCTGTCAGCAGTGC-
CAATGGGACTGTGCTGAAGAGTACAGGCAGCGGCCCTGTCTCCTCTGGGGGCCTTATG-
CAGCTGCCTACCAGCTTCACCCTCATGCCTGGTGAGTCACGAGGGGCAGGGAGAGATTC-
GTCCTTCCTGGGGCAAATCAAGCATGCTAAGAGTGTGTTTAGGGCTGGCACCAAGAGA-
ACCTCTTTCCCTGCGATCAAAGCCCCTCCTGAGACCCAGCTCCAAGCCGTGGACTCTTC-
GGAGAACTTTCAGATCTCCCTTAAGAGCAAACAAGGCCCGATCGATGTTTTCCTGT-
GCCCTGAGGAGACCGTAGGTGGGATCAGCCCTGGGAAGACCCCATCCCAGGAGGT-
CACTTCTGAGGAGGAGAACAGGGCCACTGACTCTGCCACCATAGTGTCACCACCAC-
CATCATCTCCCCCCTCATCCCTCACCACAGATCCCAGCCAGTCTCTACTCAGCCTG-
GAGCAAGAACCGCTGTTGTCCCGGATGGGCAGCCTGCGGGCTCCCGTGGACGAG-
GACCGCCTGTCCCCGCTGGTGGCGGCCGACTCGCTCCTGGAGCATGTGCGGGAG-
GACTTCTCCGGCCTCCTCCCTGAGGAGTTCATCAGCCTTTCCCCACCCCACGAGGCCCTC-
GACTACCACTTCGGCCTCGAGGAGGGCGAGGGCATCAGAGACCTCTTCGACTGT-
GACTTTGGGGACCTCACCCCCCTGGATTTCTGA

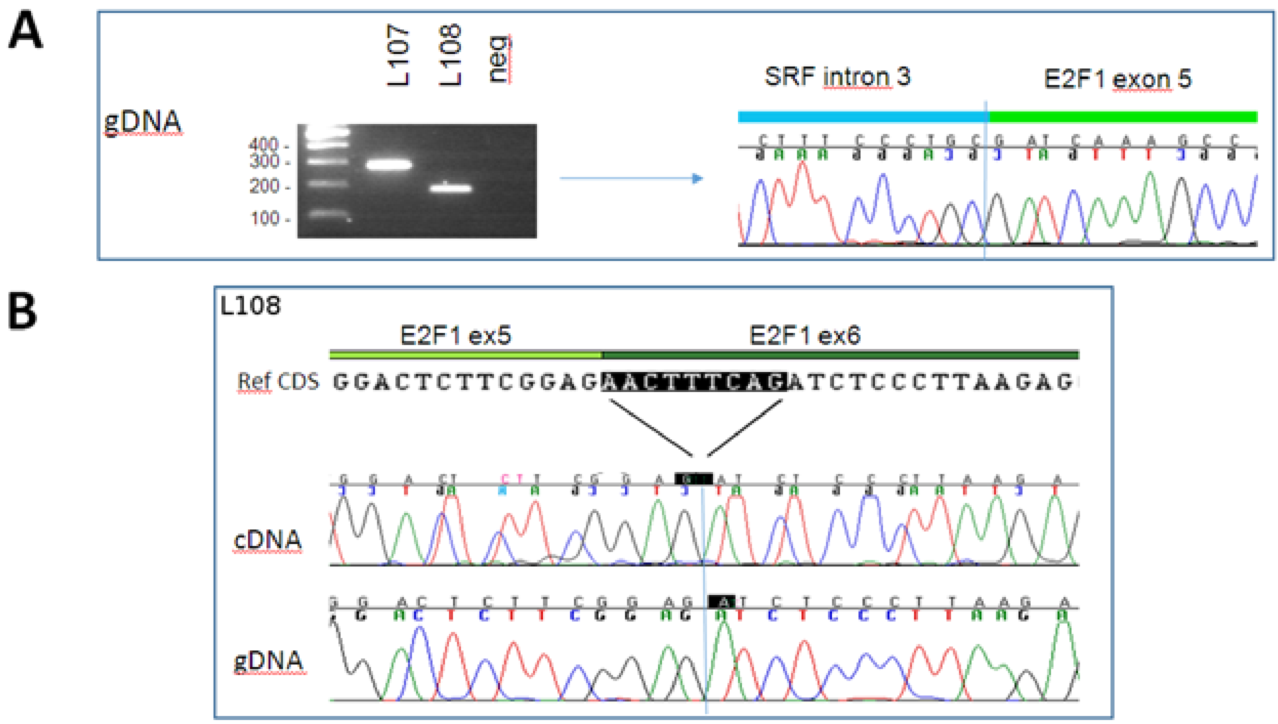

Supplementary File 2. Identification of SRF-E2F1 genomic breakpoint. A) PCR of SRF-E2F1 breakpoint performed on genomic DNA. Amplicons sizes were different between L107 and L108, while the sequence of the breakpoint (shown in the electropherogram on the right) was the same in both sample and corresponded to the one detected on cDNA. This discrepancy was due to a deletion occurred downstream the breakpoint region as highlighted in figure B. B) Two chromatograms showing the junction between exon 5 and exon 6 of E2F1 obtained from transcript (cDNA) and genomic DNA (gDNA) of L108. Focusing on this region it was possible to detect the deletion occurred downstream of the fusion breakpoint and that led to the loss of the entire intron 5 and of the first 9 nucleotides of exon6 of E2F1.

Supplementary Table 3. List of somatic mutations identified in the two SRF-E2F1 positive cases.

	Gene	Exon	cDNA	protein	type of mutation	chr position	ratio mutated allele	dbSNP	ExAc
**L107**	TYRO3	exon17	c.2145delG	p.V715fs	frameshift_deletion	15:41865665-41865665	0,25	Novel	8,60E-04
	PPP2R2B	exon1	c.58_59insAGCAGCAGCAGCAGC	p.C20delinsSSSSSC	nonframeshift_insertion	5:146258290-146258290	1,00	Novel	n
	PROCR	exon2	c.G293T	p.R98L	nonsynonymous_SNV	20:33762727-33762727	0,28	rs200377875	3,09E-03
	ZNF717	exon5	c.1727_1728insTATAAGT	p.S576_F577delinsLX	stopgain_SNV	3:75787047-75787047	0,23	Novel	n
	ZNF717	exon5	c.C734T	p.A245V	nonsynonymous_SNV	3:75788040-75788040	0,24	rs111880168	n
	FRG1	exon5	c.C427A	p.Q143K	nonsynonymous_SNV	4:190876301-190876301	0,35	rs150472183	n
	DDX47	exon9	c.C973T	p.R325X	stopgain_SNV	12:12977549-12977549	0,45	Novel	7,41E-05
	KIR2DL3	exon4	c.C662T	p.T221I	nonsynonymous_SNV	19:55255534-55255534	0,47	rs150145497	n
	KIAA1804	exon5	c.C1349A	p.A450D	nonsynonymous_SNV	1:233497836-233497836	0,26	Novel	n
	DMGDH	exon11	c.G1687T	p.G563C	nonsynonymous_SNV	5:78325854-78325854	0,49	Novel	1,66E-05
	COL4A3BP	exon5	c.349-2->T	nn	INDEL_splicing	5:74722305-74722305	0,21	Novel	7,90E-03
**L108**	LRP2	exon22	c.C3253T	p.R1085C	nonsynonymous_SNV	2:170101380-170101380	0,30	Novel	8,24E-06
	SLC43A1	exon7	c.A655G	p.I219V	nonsynonymous_SNV	11:57263541-57263541	0,48	rs202001356	1,49E-04
	HYDIN	exon48	c.G8185A	p.G2729R	nonsynonymous_SNV	16:70942584-70942584	0,26	rs199990018	3,53E-03
	CDC27	exon14	c.C1843A	p.H615N	nonsynonymous_SNV	17:45214606-45214606	0,33	rs79260965	n
	KIR2DL1	exon3	c.C272A	p.T91K	nonsynonymous_SNV	19:55284986-55284986	0,45	rs117204680	4,24E-05
	SNX7	exon2	c.G350A	p.R117K	nonsynonymous_SNV	1:99150610-99150610	0,38	rs61756174	3,37E-03
	TYSND1	exon1	c.T338C	p.L113P	nonsynonymous_SNV	10:71906005-71906005	0,60	rs145700158	9,41E-03
	EMR1	exon16	c.C2221T	p.R741C	nonsynonymous_SNV	19:6926611-6926611	0,54	rs149410886	3,30E-05
	DNAH7	exon17	c.C2198A	p.A733E	nonsynonymous_SNV	2:196834679-196834679	0,40	rs78418952	8,45E-04
	MUC22	exon3	c.T4381G	p.S1461A	nonsynonymous_SNV	6:30997589-30997589	0,37	rs11754361	n
	POP1	exon15	c.C2339T	p.S780L	nonsynonymous_SNV	8:99168559-99168559	0,46	rs149744031	7,41E-05
	SKA3	exon9	c.1120-2->T	nn	INDEL_splicing	13:21729952-21729952	0,88	rs11446085	n
